# Comparison of Adipocyte Viability After Short-Term Cryopreservation of Adipose Aspirates Through 3 Different Techniques

**DOI:** 10.1093/asjof/ojad026

**Published:** 2023-04-04

**Authors:** Polen Koçak, Naz Ünsal, Serli Canikyan, Yaren Kul, Steven R Cohen, Tunç Tiryaki

## Abstract

**Background:**

Effective cryopreservation allows for the long-term storage of living cells or tissues with the possibility of later clinical applications. Unfortunately, no successful investigations on the long-term preservation of adipose aspirates for prospective autologous fat grafting have been conducted.

**Objectives:**

In this study, we aimed to compare 3 different freezing methods to preserve adipose aspirates obtained from conventional lipoplasty to determine the optimal cryopreservation technique.

**Methods:**

To determine the optimal cryopreservation technique, hematoxylin and eosin staining, MTS assay, and Annexin assay were performed on each of the 3 groups plus a fourth control group. Group 1 served as the control, and fat tissue was analyzed immediately after adipose harvesting with no cryopreservation. For experimental Group 2, 15 mL of adipose aspirates were directly frozen at −80°C for up to 2 weeks. For experimental Group 3, 15 mL of adipose aspirates were frozen inside the adi-frosty containing 100% isopropanol and stored at −80°C for up to 2 weeks. For experimental Group 4, 15 mL of adipose aspirates were frozen with freezing solution containing 90% fetal bovine serum (v/v) and 10% dimethyl sulfoxide (v/v).

**Results:**

The results demonstrated that the experimental Group 3 had significantly more live adipocytes and greater cellular function of adipose aspirates than the experimental Groups 2 and 4.

**Conclusion:**

Cryopreservation with adi-frosty containing 100% isopropanol appears to be the best means of cryopreservation of fat.

**Level of Evidence: 3:**

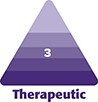

Autologous fat transplantation (AFT) is a cosmetic method used by plastic surgeons to rejuvenate some parts of the body, face, and hands due to aging. In addition, it is a procedure that is used to correct the irregularities caused by lipoplasty, such as removing the soft-tissue deficiencies caused by iatrogenic or traumatic defects.^1,2^ The simplicity of AFT and the abundance of donor areas in most patients explain why it is widely used in plastic surgery for reconstructive and aesthetic purposes.^3^ On the other hand, this process has a disadvantage such as difficulty in predicting transplant survival rates after adipose fat tissue. The reason for this unpredictable situation is the absorption of a variable part of the transplanted adipose tissue or necrosis.^4^ Survival rates varying from 30% to 80% have been recorded, and because of this uncertainty, overcorrection or retransplantation has been used to overcome the high adipose tissue absorption rates.^5–7^ Although the techniques for AFT are optimized, there may be situations that require more than one fat transfer surgery to achieve or maintain the desired therapeutic effect. When more than one fat transfer operation is needed, it creates an additional surgery for the patient along with its potential complications and adverse outcomes. The need for more than one fat transfer operation, as well as the potential improvement in regenerative and anti-aging outcomes with serial treatments, makes cryopreservation of adipose tissue for reconstructive or regenerative medical purposes an important consideration in patient management. Since overcompensation with fat grafting contributes to unnatural cosmetic results and retransplantation entails repeat surgery, cryopreservation of the remaining adipose tissue enables simple retransplantation if needed.^8^ Although there have been a number of studies on the freezing of adipose aspirates, most have been conducted at cryopreservation temperatures that were on the high side (between +1°C and −18°C) and as a consequence, did not have satisfactory results.^9,10^ The survival rate of adipocytes being cryopreserved is critical when adipose tissue is reused after storage, since obviously viable cells will have a large effect on adipose tissue engraftment after reinjection. Both plastic surgeons and patients have expressed a deep desire to retain adipose tissue aspirates for future applications if an appropriate cryopreservation procedure could be developed.

In this study, we compare 3 different freezing methods by performing with the measurement of the volume of the lipid layer, measurement of the number of adipocytes that survived, hematoxylin and eosin (H&E) staining, MTS assay, cell-cycle assay, and Annexin assay in order to determine the optimal means of cryopreservation of adipose aspirates obtained from conventional lipoplasty. Experimental methods are shown schematically in the Supplemental Figure.

## METHODS

### Procedure for Liposuction

The operations were performed under local anesthesia with or without sedation, and the grafts were harvested primarily from the lateral thighs, and rarely from the lower back and abdomen. After the donor site was infiltrated with a solution of saline and epinephrine, a 2.4 mm cannula and a traditional Coleman injection cannula were used, for the aspiration and fat transfer, respectively.^5^

### Procedure for Cryopreservation

Several freezing protocols were attempted during our study. Group 1 served as the control and 15 mL of freshly liposuctioned fat was subjected as 4 different groups. The experimental samples from each group were frozen and thawed. For experimental Group 2, 15 mL of adipose aspirates were directly frozen at −80°C for up to 2 weeks. For experimental Group 3, 15 mL of adipose aspirates were frozen inside the adi-frosty containing 100% isopropanol and stored at −80°C for up to 2 weeks. For experimental Group 4, 15 mL of adipose aspirates were frozen with a freezing solution that contained 90% fetal bovine serum (FBS; Gibco, Thermo Fisher Scientific, Waltham, MA) (v/v) and 10% dimethyl sulfoxide (DMSO; Sigma-Aldrich, St Louis, MO) (v/v). Groups were subjected to the following measurements after defreezing of the sample at the waterbath: volume of lipid layer after centrifugation, number of viable adipocytes, mitochondrial activity, Annexin assay, Muse (Luminex, Austin, TX) count of regenerative cell numbers and types, and H&E staining.^9^

### Measurement of the Number of Adipocytes That Survived

After centrifuging the adipose tissues for 3 min at 1610 x g, 10 μL of adipose tissue in the middle layer of each sample group was taken and stained with 10 μL of trypan blue (Sigma-Aldrich, St Louis, MO). Using the cell-counting chamber, the number of live cells in a 1 μL sample was counted. Trypan blue does not stain the living cells. The number of adipocytes was counted with the Invitrogen Countess II Automated Cell Counter (Waltham, MA) in the 50–200 μm cell size range. In the live/dead assay performed with cell counter, "Live" cells are circled in green and "Dead" cells are circled in red on the screen by the system of device.^11^

### Muse Count of Regenerative Cells After Enzymatic Digestion

Cell counting was done for each of the 4 sample groups after enzymatic digestion with the Muse cell-cycle assay kit (Luminex). 20 μL of each cell suspension was added to 1.5 mL of Eppendorf tubes (Hamburg, Germany), and 780 μL of count viability reagent was added to these Eppendorf tubes. Detections were performed using Muse Cell Analyzer (Luminex).

### Annexin Assay

Three experimental groups as well as the control were centrifuged. After centrifuging the adipose tissues for 3 min at 1610 x g, 10 mL of adipose tissue from the middle layer of each sample group was collected, and Type II collagenase solution was added and mixed with each sample group for the enzymatic digestion. After histolysis for 1 h in an incubator at 37°C, the treated tissues were centrifuged for 10 min at 1610 x g and the supernatant was removed. All sample groups were mixed with 2 mL of Dulbecco's modified Eagle's medium (DMEM; Gibco, Thermo Fisher Scientific). Apoptotic effects of freezing methods were examined by the Annexin V Detection Kit (Abcam, Cambridge, UK) in accordance with the protocol of manufacture. Samples were incubated with Annexin V fluorescein isothiocyanate and propidium iodide (PI) phycoerythrin (PE) conjugated antibodies. Detections were performed using FACS Calibur flow cytometry (BD Biosciences, San Jose, CA). Analysis of the data was executed via the CellQuest Pro program (BD Biosciences).^11^

### Measurement of the Volume of the Lipid Layer After Centrifugation

For each of the 3 experimental groups as well as the control, the volume of the supernatant lipid layer was determined after centrifugation at 1610 x g for 3 min using a centrifuge, and the degree of adipocyte damage was assessed indirectly via this method based on an increase in oil volume that occurs with the increase in adipocyte damage in lipoaspirates. Aspirated fat tissue was divided into different layers from top to bottom after centrifugation: oil, fat, and fluid. After centrifugation, the oil ratio was calculated as follows: oil ratio = (oil volume)/[(oil volume) + (fat volume)].

### Measurement of Mitochondrial Activity

The MTS assay (3-(4,5-dimethylthiazol-2-yl)-5-(3-carboxymethoxyphenyl)-2-(4-sulfophenyl)-2H-tetrazolium) was used to determine the adipocytes’ mitochondrial function. In this study, the effect of different types of cryopreservation on mitochondrial activity was checked. MTS reagent is a tetrazolium inner salt used in proliferation and chemosensitivity assays to determine the number of viable cells. Cells convert MTS into a colored formazan substance that is soluble in tissue culture medium. In metabolically active cells, NAD(*P*)H-dependent dehydrogenase enzymes are thought to carry out this conversion. After centrifuging the adipose tissues for 3 min at 1610 x g, 200 μL adipose tissue from the middle layer of the sample group was collected and placed in a 15 mL Falcon tube. 500 mL of DMEM was mixed with 200 μL of MTS solution (Abcam). This mixture was added to adipose tissue and incubated at 37°C for 1 h. After incubation, 100 mL of adipose tissue samples from the falcon tube were transferred on 96-well plate, and the plate was read at 490 nm in the Elisa Plate Reader.^12^

### Hematoxylin and Eosin (H&E) Staining

Finally, the histologic analysis of the fresh or cryopreserved adipose aspirates (about 2-3 g) was performed to see the effect of these 3 different techniques on the pattern, shape, and structure of cells in a tissue sample. Fresh adipose tissues embedded in optimal cutting temperature (OCT) compound in cryomolds and blocks frozen overnight at −180°C. Tissue blocks were cut into 20 μm thick cryostat sections and mounted on superfrost plus slides and then all slides were incubated at −180°C until stain. Slides were incubated at room temperature for 30 min and then fixed in a 4% paraformaldehyde solution for 30 min before staining. Then rinsed in phosphate-buffered saline and proceeded to 100%, 90%, 80%, and 70% ethanol series for 10 min. Then sections were brought to tap water and stained with Mayer's hematoxylin solution for 2 min. The sections were then washed in tap water and stained with Eosin stain for 1 min. Afterwards, sections were dehydrated in 70%, 80%, 90%, and 100% ethanol for each 10 min and then cleared in xylene for 30 min. The slides were mounted with mounting medium, and examination was performed using the light microscope.^13^

### Statistical Analysis

A mean and standard deviation were used to define the characteristics of the population. A Mann–Whitney test was used for statistical analysis. Statistical significance was defined as an alpha value of *P* < .05. GraphPad Prism version 7.00 for Windows, GraphPad Software (La Jolla, CA) was used for all statistical analyses.

## RESULTS

**Figure 1.**
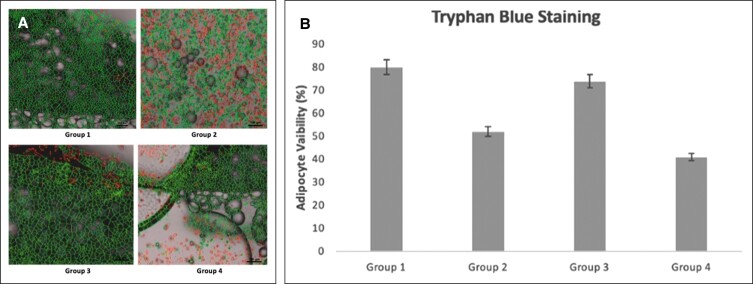
(A) Cell viability results after freeze thawing. Green circles refer to living cells. Red circles refer to dead cells. (B) Graph of trypan blue assay results for the adipocytes viability (%) with each group. Each data set represents the percentage expression ± standard error from 3 samples, and images are representatives of 3 independent samples (*n* = 3).

Living adipocytes for each group were stained with trypan blue dye, and 10 μL samples were prepared for each group and placed in the cell-counting chamber and viable cells were observed. According to this counting, it was found that 80% cells were counted in Group 1, the control, 52% cells in Group 2, which was directly frozen at −80°C for up to 2 weeks, 74% cells in Group 3, which were frozen inside the adi-frosty containing 100% isopropanol, 41% cells in Group 4 were frozen with a freezing solution containing 90% FBS (v/v) and 10% DMSO (v/v). When comparing the 4 groups, the adipocyte viability in Group 3 was statistically higher than Groups 2 and 4 and almost the same as the noncryopreserved control, as shown in Figure 1.

**Figure 2.**
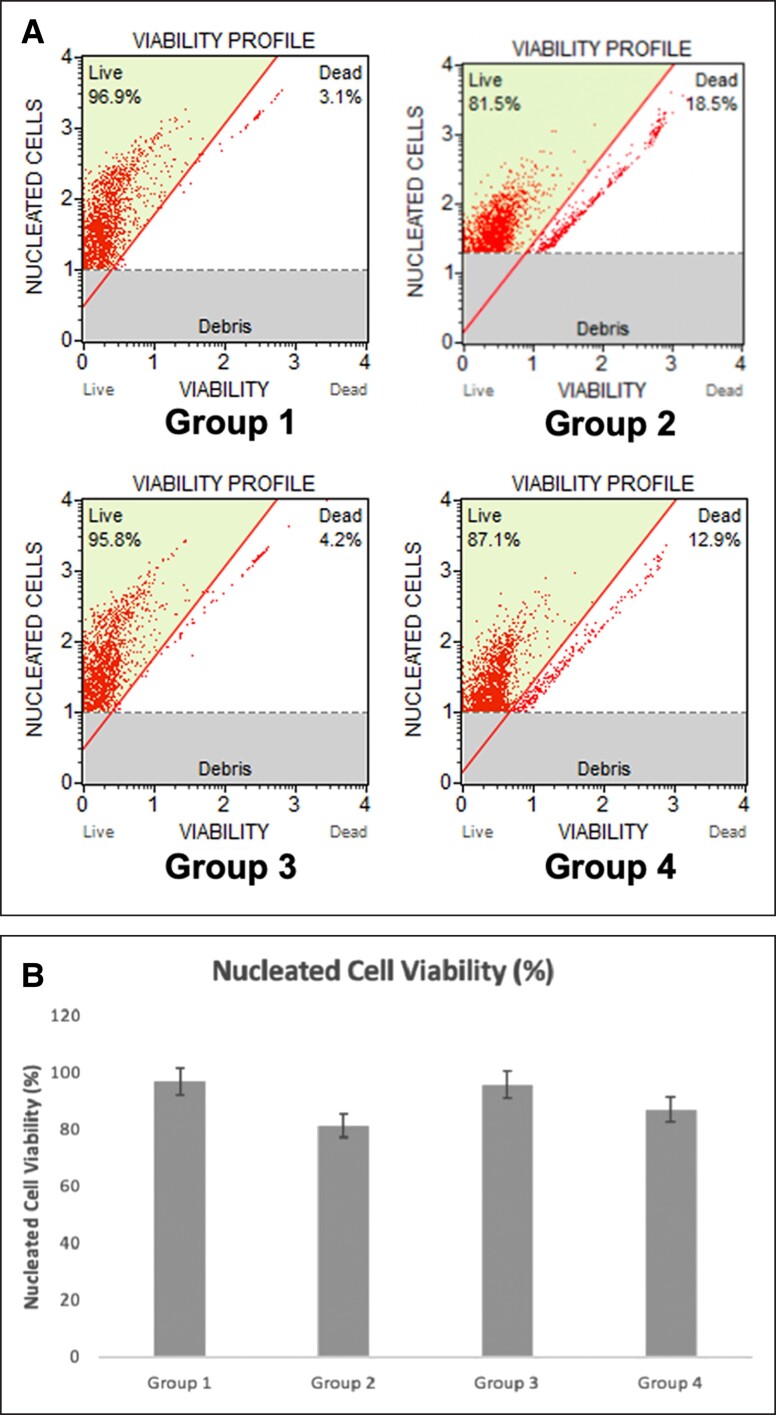
(A) Nucleated cell viability profiles were examined by Muse cell-cycle assay for each group and separated as live and death with percentages. (B) Graph of nucleated cell viability with percentages for Groups 1-4. Each data set represents the percentage expression ± standard error from 3 samples, and images are representatives of 3 independent samples (*n* = 3).

By using the Muse cell-cycle assay, nucleated cell viability profile is obtained. Group 1 control cells were measured directly without any freezing technique and had a cell viability of 96.9%, which is the highest percentage of viable cells (Figure 2). Group 2, which was frozen directly at −80°C, demonstrated a cell viability percentage of 81.5%. In Group 3, cells were frozen with adi-frosty, and the percentage of live cells was found to be 95.8%, nearly equivalent to the noncryopreserved cells (Figure 1A). Group 4 cells were frozen with a freezing solution containing 90% FBS (v/v) and 10% DMSO (v/v) inside adi-frosty and its live cell percentage was 87.1% (Figure 2).

**Figure 3.**
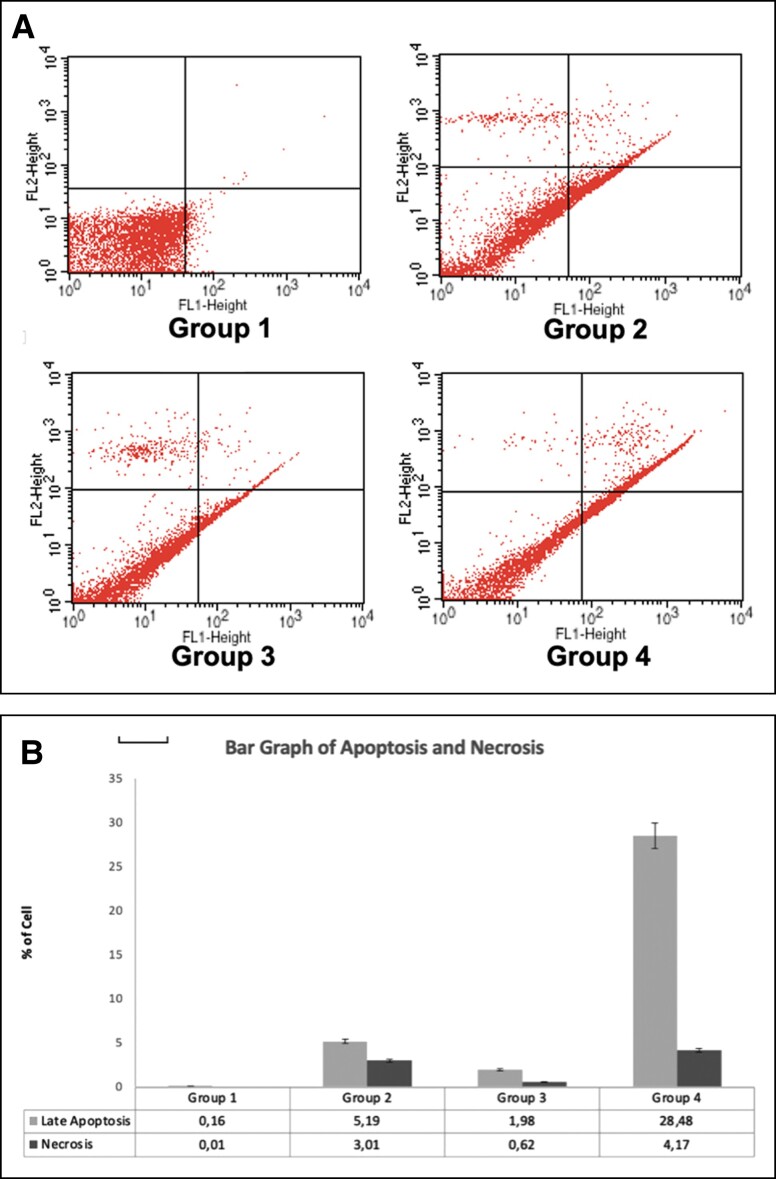
(A) Four quadrant images were observed by flow cytometry analysis for each group. (B) Graph represents percentages of late apoptosis and necrosis for each group. Each data set represents the percentage expression ± standard error from 3 samples, and images are representatives of 3 independent samples (*n* = 3).

Annexin V staining was performed in order to indicate whether cells were viable or had undergone apoptosis or necrosis. Late apoptosis was seen mostly in Group 4 with 28.8%. The highest number of viable cells were observed in Group 1, which was the control group. Moreover, no significant apoptotic cell numbers were detected in Group 3 compared to the control group. Group 3 had the second highest number of dead cells (5.19%) after Group 4 (Figure 3).

**Figure 4.**
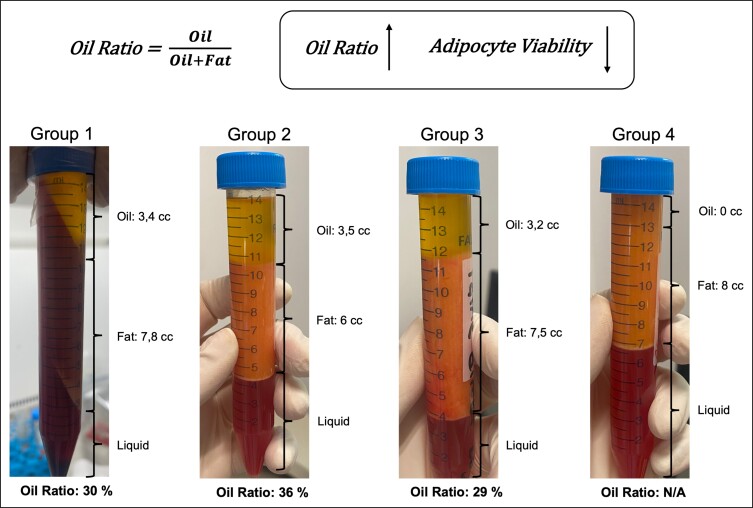
Analysis of adipocytes degeneration in lipoaspirates by centrifugation and aspirated fat tissue was divided into different layers from top to bottom after centrifugation: oil, fat, and fluid. The oil ratio was calculated as follows: oil ratio = (oil volume)/[(oil volume) + (fat volume)]. Each data set represents the percentage expression ± standard error from 3 samples, and images are representatives of 3 independent samples (*n* = 3).

When the oil rates are compared, it is seen that there is no significant difference between the ratio of noncryopreserved adipose and the oil ratios in Groups 2 and 3. When Group 4 is examined, it is seen that the oil ratio was zero compared to the other groups after 2 weeks of freezing (Figure 4).

**Figure 5.**
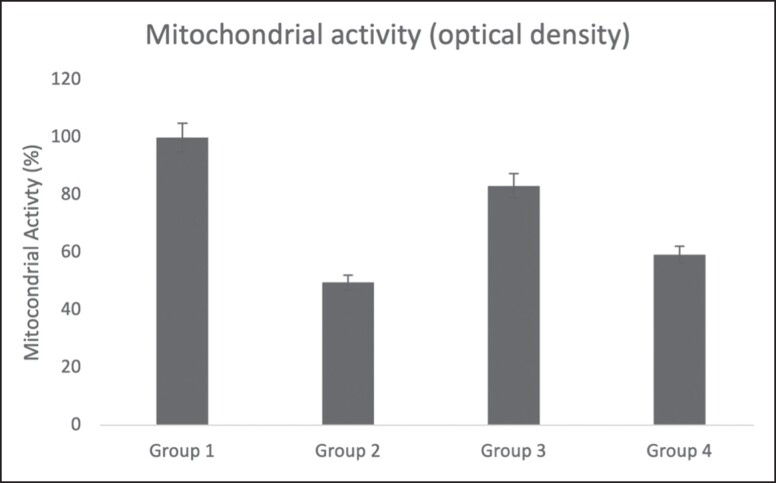
Measurement of mitochondrial activity by MTS (3-(4,5-dimethylthiazol-2-yl)-5-(3-carboxymethoxyphenyl)-2-(4-sulfophenyl)-2H-tetrazolium) assay. Each data set represents the percentage expression ± standard error from 3 samples and images are representatives of 3 independent samples (*n* = 3).

The optical density (OD) of the upper layer was measured in the MTS assay after a 1 h reaction between the MTS reagent and the sample groups. The OD was 0.751 to 0.621 (mean, 0.686) in Group 1 used immediately after adipose harvesting, 0.366 to 0.315 (mean, 0.340) in Group 2 was directly frozen at −80°C for up to 2 weeks, 0.513 to 0.629 (mean, 0.571) in Group 3 were frozen inside the adi-frosty, 0.384 to 0.429 (mean, 0.406) in Group 4 were frozen with freezing solutions containing 90% FBS (v/v) and 10% DMSO (v/v) and inside the adi-frosty. When Group 1 was compared with other groups, Group 3 mitochondrial activity was found to be the highest of the other 2 experimental groups, as shown in the Supplemental Table and Figure 5.

**Figure 6.**
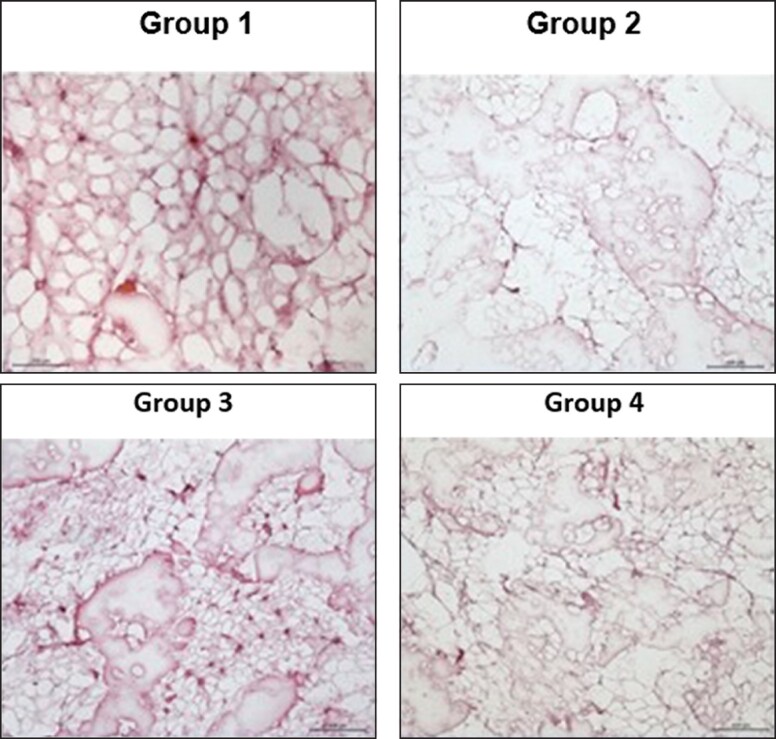
Hematoxylin and eosin staining: Group 1—fat tissue after harvesting; Group 2—directly put the fat tissue into −80°C and checked after 2 weeks; Group 3—put the fat tissue into the Adi-Frosty and then −80°C and checked after 2 weeks; and Group 4—adipocytes were frozen with freezing medium and stored at −80°C and checked after 2 weeks. Each data set represents the percentage expression ± standard error from 3 samples, and images are representatives of 3 independent samples (*n* = 3).

The aim of the H&E staining is to demonstrate the effect of freeze thawing on the fat integrity. As a result of the study, the collagen fibers and blood vessels of the adipose tissue were less damaged after freezing and thawing in Group 3 when compared with Groups 2 and 4, and all groups were compared with fresh adipose tissue as a control group. In other words, when compared with Groups 2 and 4, in Group 3, larger intercellular spaces with abundant collagen fibers were observed and more blood vessels were detected in intercellular spaces, as shown in H&E staining results (Figure 6).

## DISCUSSION

Accurate storage of harvested adipose tissue prevents the need for an additional liposuction step, thus increasing patient’s acceptability and also minimizing pain, saving time, and money.^14^ The types of cryoprotectant, cooling rate, and thawing rate are the crucial variables that affect how stem cells freeze. The optimization of these parameters on adipose derived stem cells has been the subject of several studies.^13–16^ However, because of the complex compositon, each tissue has a distinct optimum freezing temperature and mode, and cryopreservation of composite tissues is more difficult than homogeneous cell population.^17^ In the literature, there are many studies on the cryopreservation of adipose tissue according to various protocols with or without the usage of cryoprotectants.^18,19^ According to the experimental results, cryoprotectant usage has some advantages for cell viability following thawing.^20^ In contrast to the lack of a cryoprotective agent, the outcomes of DMSO and FBS contained freezing medium combination, one of the most common cryoprotective agents, enhanced cell survival, and the retention volume of adipose tissue.^21–23^ Similar to this, ASCs that were cryopreserved with DMSO and polyvinylpyrrolidone (PVP) and kept for 2 weeks at −196°C kept their post-thaw viability as well as their osteogenic and adipogenic potentials. However, DMSO and FBS combination carries the risk of toxicity, and because of the complexity and structural integrity of tissue compared with isolated cells, it may be challenging to thoroughly elute DMSO.^24,25^ For instance, it has been demonstrated that exposure to DMSO causes significant alterations in stem-cell markers, protein content, and functionality.^26^ Considering the disadvantages of cryoprotectant agents, it is important to develop a DMSO-free, serum-free, and even xeno-free cryoprotection methods for cryopreservation of adipose cells and tissues. Cooling rate is another crucial topic for tissue cryopreservation process. At high cooling rates, water could not eliminate quickly and leads to intracellular ice formation, cell membrane rupture, and cryoinjury. The optimal cooling rate for cell survival should be slow enough to reduce intracellular ice formation but fast enough to minimize the solution effects.^27^ However, the majority of the studies only focused at one of the aforementioned characteristics and mainly ignored the impact of long-term freeze storage times. Provided long-term preservation of the aspirates is enabled, adipose aspirates acquired after standard lipoplasty might be a helpful source for future AFT or possibly for tissue engineering. It is unfortunate that there is now no best strategy for the long-term preservation of adipose tissues, and research that attempt to find such a way are few and not particularly productive.^28^ That's why in this study we aimed to compare the survival rates of freshly liposuctioned fat and cryopreserved fat using 3 different freezing methods including direct freezing, freezing in the adi-frosty containing 100% isopropanol and freezing solution with 90% FBS (v/v) and 10% DMSO (v/v). Our ideal cryopreservation strategy retained much more live adipocytes and greater cellular function in adipose aspirates than a straightforward cryopreservation procedure.

Survival rates of adipocytes were determined by live cell counting with trypan blue staining and after enzymatic digestion with Muse cell-cycle assay kit. The measurement of mitochondrial activity, which is a way of assessing cell functionality was performed using the MTS assay. Annexin V was used to detect apopototic cells, and H&E staining was done for the observation of cell morphology.

Live cell counting was performed by staining adipocytes with using trypan blue staining, and the percentage of viable cells was determined for each group. When looking at Figure 1A, it is seen that live cells are shown as green circles and dead cells are shown as red circles. When we look at the graph in Figure 1B, when compared with Group 1, it is seen that the highest cell viability is in the adipocytes frozen with the Group 3 method, which used adi-frosty and100% isopropanol. Less cell viability was seen in the adipocyte samples, which were frozen directly to −80°C and frozen with 90% FBS (v/v) and 10% DMSO (v/v) freezing solution, compared to the other 2 groups.

After trypan blue staining, in order to determine cell concentration and confirm viability, the Muse cell-cycle assay was also applied. With the Muse cell-cycle assay, nucleated cell viability profiles were obtained (Figure 2). With this method, Group 3 had the closest viability to the control group with a rate of 95.8%. Although the other experimental groups had >80% of viable cells, highest counts of dead cells were observed in Group 2 as expected, which were frozen directly at −80°C. From this study, freezing cells with adi-frosty in 100% isopropanol is more effective than freezing cells with freezing medium (1% DMSO), likely because some degree of toxicity of DMSO affects the viability of cells while freezing.

The use of Annexin V labeling was carried out in order to determine whether cells were alive or were undergoing late apoptosis or necrosis. No significant apoptotic effect was found in any of the 3 cryopreservation methods. However, since DMSO is a toxic solvent, the highest apoptotic effect was observed in Group 4 even though Group 4 was frozen with adi-frosty. The greatest number of viable cells were found in Group 1, which functioned as the control group, and there was no significant difference in the amount of apoptotic cells found in Group 3 when compared with the control group. Therefore, freezing cells with adi-frosty in 100% isopropanol keeps cells alive with similar functional qualities as the noncryopreserved control group and was found to be the most effective method among the 3 procedures for cryopreservation.

For the sensitive measurement of live cells, the MTS assay employs a colorimetric technique. It is based on a single reagent that is ready to use. Cell proliferation, viability, and cytotoxicity are all assessed using the MTS assay. The MTS assay procedure relies on live mammalian cells (and also cells from other species) reducing the MTS tetrazolium molecule to produce a colored formazan dye that is soluble in cell culture medium. In metabolically active cells, NAD(*P*)H-dependent dehydrogenase enzymes are assumed to carry out this conversion. The absorbance of the formazan dye is measured at 490 to 500 nm to determine its concentration.^29^ In this study, the absorbance of formazan dye assessed by MTS assay before freezing adipocytes (Group 1 control) upon sampling was reported to be 0.686. Since this group was the control group, cell viability was expected to be the highest and close to 100%. Accordingly, the results of other cryopreservation methods were compared to the noncryopreserved control Group 1. Looking at Figure 5, it is seen that the cell viability closest to the control group is Group 3. Adipocytes in Group 3 were frozen inside the adi-frosty, which contained 100% isopropanol and stored at −80°C for up to 2 weeks. Adipocyte samples frozen directly to −80°C were Group 2 and as expected had the least viability as a result of the MTS experiment. The reason for this is that when the cells are frozen rapidly, the salt concentration in the environment suddenly increases and the cells shrink and die as a result of the increase in osmotic pressure. During cooling, the salt in the environment begins to emit heat inversely proportional to the cooling rate, and this heat damages the cells (thermal damage). In Group 4, adipose aspirates were frozen with a freezing solution that contained 90% FBS (v/v) and 10% DMSO (v/v). Looking at the graph in Figure 5, it is seen that it gives 10% more viability than Group 2, but less viability compared to the other 2 groups. Compared to Group 2, although both of them were frozen in the adi-frosty, less cell viability was detected for the samples in Group 4, even though an extra freezing solution (90% FBS [v/v] and 10% DMSO [v/v]) was used. This is thought to be due to the toxic effect of DMSO, which is a cryoprotectant. In the literature, studies have shown that DMSO has a toxic effect on some cell lines even at low doses.^12^ Therefore, when 3 different freezing methods for adipocytes are compared, it can be said that the best method is the method in Group 3 because this group gave the closest results to adipocyte viability in the samples taken on the first day.

According to Boschert et al,^30^ centrifugation at >100 × *g* can affect adipocytes’ viability; however, we recently discovered that centrifugation at 2063 × *g* did increased the oil portion in lipoaspirate groups, but did not significantly injure adipocytes or increase oil volume. According to Figure 4, the oil ratios in the first 3 groups were similar, whereas in Group 4, the oil ratio was zero. This is thought to be due to the DMSO in the freezing solution used for freezing.

Lastly, H&E staining is important to analyze the morphology of the tissue samples after different types of freezing techniques. As a result, in Group 3, larger intercellular spaces with abundant collagen fibers^27^ were detected when compared with Groups 2 and 4, and more blood vessels in intercellular spaces were detected, which in immunohistochemical characterization suggests that Group 3 had the highest efficiency in freezing compared to the others.

In conclusion, different cryopreservation techniques have marked differences in their ability to maintain the cell viability of fat cells as well as regenerative cells found within adipose tissue. In cryopreservation, adi-frosty and 100% isopropanol appeared to preserve the maximum functionality as well as viability compared to other freezing techniques.

In this study, we cryopreserved adipose tissue for 2 weeks. The freezing times applied in previous studies on adipose tissue cryopreservation ranged from days to 2 months.^18–23^ In this scope, to determine whether the duration of cryopreservation will affect the viability of cells and tissues, long-term studies must be conducted. Comparison studies of short- and long-term cryopreserved fat grafts with control groups (noncryopreserved fresh adipose tissue) are ongoing, and the relevant results will be the subject of our next publication. Furthermore, in vitro, in vivo, and clinical future studies are needed to further prove the safety of adipose tissue cryopreservation using this method and to optimize its reliability. In this direction, in our ongoing prospective study, both quantitative and qualitative clinical results and comparisons evaluating the clinical effectiveness of the method will be shared in our future publications.

## CONCLUSIONS

In this study, we present the comparisons of 3 different freezing methods by performing with the measurement of the volume of the lipid layer, measurement of the number of adipocytes that survived, H&E staining, MTS assay, cell-cycle assay, and Annexin assay in order to determine the optimal means of cryopreservation of adipose aspirates obtained from conventional lipoplasty. Results demonstrated that the technique called adi-frosty was found to have the maximum functionality as well as viability compared to other freezing techniques. Moreover, we have ongoing studies focusing on the improvement of the freezing of adipose tissue in clinical further applications for long periods of time.

## Supplemental Material

This article contains supplemental material located online at www.asjopenforum.com.

## Supplementary Material

ojad026_Supplementary_Data
