# Supplementary material for: Comparison of Adipocyte Viability After Short-Term Cryopreservation of Adipose Aspirates Through 3 Different Techniques
Source: Aesthet Surg J Open Forum. 2023 Apr 4;5:ojad026. doi: 10.1093/asjof/ojad026 (PMC10174199; doi:10.1093/asjof/ojad026)
Supplement: ojad026_Supplementary_Data [file ojad026_supplementary_data.zip › 22-0123_Supplemental Table.docx]

**Supplemental Table:** Results of MTS

| Characteristic | Group 1 (control) | Group 2 | Group 3 | Group 4 |
| --- | --- | --- | --- | --- |
| Average absorbance value | 0.610333 | 0.320333 | 0.572 | 0.372333 |
|  | 0.709 | 0.3365 | 0.524 | 0.31 |
|  | 0.734667 | 0.334333 | 0.514 | 0.421667 |
| Cell viability | 100 | 52.48498 | 93.71928 | 61.00492 |
|  | 100 | 47.46121 | 73.90691 | 43.72355 |
|  | 100 | 45.50817 | 69.9637 | 57.39564 |
