# Supplementary material for: Comparison of Adipocyte Viability After Short-Term Cryopreservation of Adipose Aspirates Through 3 Different Techniques
Source: Aesthet Surg J Open Forum. 2023 Apr 4;5:ojad026. doi: 10.1093/asjof/ojad026 (PMC10174199; doi:10.1093/asjof/ojad026)
Supplement: ojad026_Supplementary_Data [file ojad026_supplementary_data.zip › 22-0123_Supplemental Figure Legend.docx]

**Supplemental Figure Legend**

**Supplemental Figure.** Schematic representation of experimental methods. Created with BioRender.com
